# Oxidative stress modulation in alcohol-related liver disease: From chinese botanical drugs to exercise-based interventions

**DOI:** 10.3389/fphar.2025.1516603

**Published:** 2025-04-25

**Authors:** Yuting Zhu, Yuqing Jia, Enming Zhang

**Affiliations:** School of Sports Medicine and Physical Therapy, Beijing Sport University, Beijing, China

**Keywords:** alcohol-related liver disease, Chinese botanical drugs, Chinese herb medicine, oxidative stress, mechanism

## Abstract

Alcohol-related liver disease (ALD) is a chronic liver injury caused by long-term excessive alcohol consumption, with complex and multifaceted pathological mechanisms. Research indicates that oxidative stress (OS), inflammatory responses, and lipid metabolic disturbances induced by alcohol and its metabolites are primary contributors to hepatocyte injury, positioning OS as a key target in ALD treatment. The main non-pharmacological treatment for ALD is alcohol abstinence, while medical treatment primarily relies on Western pharmacological interventions. However, recent research has increasingly highlighted the potential of Chinese botanical drugs in improving histological features and modulating signaling pathways associated with OS in ALD, underscoring the therapeutic potential of traditional Chinese herb medicine. Despite these promising findings, the precise mechanisms and effects of these extracts remain incompletely understood, and potential side effects must be considered. Therefore, a comprehensive analysis of herbal extracts with therapeutic effects is essential to optimize clinical administration and ensure safe, effective treatment. This review focuses on OS as a central theme, categorizing Chinese botanical drugs into six major groups—flavonoids, polyphenols, terpenoids, alkaloids, saponins, and anthraquinones—all widely used in traditional Chinese herb medicine. The review provides an overview of their botanical characteristics and therapeutic actions in the context of ALD, offering insights into OS regulation and exploring their potential as treatments for ALD. Notably, physical exercise shares overlapping mechanisms with botanical drugs in regulating OS. Combining two strategies could offer a promising integrative treatment for ALD, though further research is needed to confirm their synergistic benefits and optimize clinical applications.

## 1 Introduction

Excessive alcohol consumption is a major global health issue, with ALD accounting for a significant proportion of alcohol-related fatalities (Chen et al., 2023). ALD is a spectrum of liver diseases caused by chronic alcohol exposure (Jeon and Carr, 2020). It ranges from alcoholic fatty liver to alcoholic hepatitis (AH), which can progress to liver fibrosis or cirrhosis. In severe cases, it could be hepatocellular carcinoma (HCC) (Gan et al., 2025). These conditions are often accompanied by metabolic disturbances, mitochondrial damage, lipid homeostasis disruption, OS, and cell death. The development of ALD is multifaceted, shaped by complex interactions among genetic, environmental, and metabolic factors (Mackowiak et al., 2024; Malnick et al., 2022). These factors include the quantity and duration of alcohol consumption, the type of alcohol, drinking patterns, gender, ethnicity, obesity, hepatitis virus infection, genetic predisposition, and nutritional status (Pekarska and Parker, 2023; Yan et al., 2023). ALD progression is marked by the accumulation of lipid droplets (LD) in hepatocytes and oxidative damage caused by alcohol metabolism, which collectively contribute to liver inflammation and fibrosis (Lackner and Tiniakos, 2019). Given the widespread prevalence of excessive alcohol consumption (Hernández-Évole et al., 2024), ALD has emerged as a leading cause of chronic liver disease, highlighting the urgent need for effective treatment options. A deeper understanding of the intricate mechanisms underlying ALD is essential for identifying therapeutic targets, as its pathogenesis remains incompletely understood. Despite advancements in research on the disease’s pathophysiological processes, current treatment options beyond alcohol cessation remain inadequate (Stickel et al., 2017). The main current treatments for the different stages of alcoholic liver disease include long-term alcohol abstinence, nutritional therapy, management of alcohol withdrawal syndrome (AWS), Alcoholics Anonymous group meetings, psychological support, relapse prevention medications, hormone-related therapies, targeted therapies, mesenchymal stem cell treatment, and liver transplantation (Table 1).

**TABLE 1:** Current Drugs and Therapies in different stages of ALD.

General consideration	Long-term alcohol abstinence	High protein and low-fat diets
	Nutritional therapy
		Vitamin A, B, C, K, and folic acid
		Minerals (selenium, zinc, copper and magnesium)
	Alcohol withdrawal syndrome (AWS)	Symptomatic treatment
Relapse prevention	Alcoholics Anonymous group meetings	
	Psychological support	
	Alcohol withdrawal drugs	Disulfiram
		Acamprosate
		Baclofen
		Naltrexone
		Nalmefene
		Topiramate
		Gabapentin
		Ondansetron
		Sertraline
		Sodium oxybate
		Varenicline
Alcoholic Hepatitis	Hormone Related Therapies	Corticosteroids
		Propylthiouracil
		Pentoxifylline
		Antioxidants
		Losartan
		Prednisolone
Advanced-stages of ALD	Targeted Therapies	TNF Receptor Superfamily Target Therapies
		Antioxidant Signal Targeting Therapies
		Targeting the Inhibition of Hepatocyte Apoptosis
		MicroRNAs Targeted Therapies
		Gut-liver Axis Targeting
	Pluripotent Mesenchymal Stem Cells	
End-stages of ALD	Liver Transplantation	

The next stage should also adhere to the treatment principles of the previous stage. General Consideration and Relapse prevention should be applied to the whole treatment of ALD, especially in the stages of alcoholic fatty liver and early hepatitis. Hormonal therapy and targeted therapy may be considered when the condition advances to late-stage hepatitis, fibrosis, or cirrhosis. At the stages of liver failure and liver cancer, liver transplantation surgery is typically the optimal choice.

Recently, traditional Chinese herb medicines have shown potential in treating ALD (Teschke et al., 2019). While several botanical drugs have been used clinically to alleviate alcohol-induced liver damage and prevent its progression to cirrhosis or liver cancer, the exact mechanisms by which they regulate hepatocyte function remain poorly understood, raising concerns about potential side effects (Han et al., 2023). Therefore, a comprehensive analysis of Chinese herb medicine that mitigate alcohol-induced liver damage through the modulation of OS is crucial for informing their clinical use. Meanwhile, the role of exercise rehabilitation medicine in the treatment of metabolic diseases has become increasingly prominent (Barnes et al., 2010). Findings indicate that sustained and regular physical activity can enhance liver function by regulating oxidative stress, lipid metabolism, and inflammation (Vella and Cameron-Smith, 2010). Therefore, the potential synergistic effects between plant-derived compounds and exercise-induced metabolic adaptations require systematic investigation. This review not only elucidates the evidence-based mechanisms by which phytomedicines treat ALD through antioxidative stress, but also explores the potential for synergistic therapy combining exercise interventions with phytomedicine. It provides theoretical support for the application of exercise rehabilitation medicine in liver disease management.

## 2 Survey methodology

Initially, we conducted searches using broad keywords, but found that directly using terms such as “herb” or “Chinese herbal extracts” could not accurately retrieve relevant literature. Therefore, we optimized our search strategy by categorizing the botanical drugs into metabolites such as “flavonoids,” “polyphenols,” “terpenoids,” “alkaloids,” “saponins,” and “anthraquinones,” and combining these with more specific keywords like “ALD,” “alcohol-related liver disease,” “alcoholic liver disease,” “oxidative stress,” and “ROS” to improve the search accuracy. Additionally, we included specific names of Chinese botanical drugs in the search and conducted a second round of searches. We also reviewed the reference lists of relevant papers to retrospectively identify any missing studies, ensuring the comprehensiveness of the search. Based on this, we assessed the accessibility and relevance of the literature, selecting studies directly related to the topic, while excluding those that did not meet the criteria. Ultimately, 45 animal and clinical studies were included for the review (Table 2).

**TABLE 2:** Traditional Chinese botanical drugs for the treatment of ALD and the regulation of oxidative stress effects.

Classification	Active metabolite	Source	Model	Treatment/Dose	Hepatotoxicity	Mechanisms	Regulatory target molecules	Reference
			Animal model/Cell model	Animal model/Cell model				
Flavonoids	Quercetin	Quercus aliena Biume	Chronic alcohol-fed Sprague–Dawley rats (4.0 g/kg per day for 90 days)	Quercetin (100 mg/kg per day) for 90 days	↓AST; ↓ALT ↓TG; ↓TC	Antioxidation: ↑GSH ↑Mn-SOD; ↑GPx ↓ROS; ↓MDA	—	Tang et al. (2012)
			Acute alcohol-fed Human hepatocytes (100 mM, 24 h)	Quercetin (10,25,50,100,200 M) for 5 or 24 h	—	Antioxidation: ↑GSH ↓MDA; ↑HO-1	Nrf2 MAPK	Yao et al. (2007)
			Chronic alcohol-fed Sprague–Dawley rats (4.0 g/kg per day for 90 days)	Quercetin (100 mg/kg.bw per day) for 90 days	—	Antioxidation: ↑HO-1 CYP2E1↓	—	Tang et al. (2013)
			Acute alcohol-fed Human hepatocytes (100 mM, 24 h)	Quercetin (10,25,50,100,200 M) for 24 h				
			Acute alcohol-fed wild-type AB zebrafish (350 mM 2% ethanol for 32 h)	Quercetin pre-treatment 48 h at 100 μM, 50 μM, 25 μM	↓ALT; ↓AST ↓γGT; ↓TG ↓TC	Antioxidation: ↓ROS ↓MDA; ↑SOD ↑CAT ↑GSH; ↑HO-1	Nrf2	Zhao et al., 2021
			Acute alcohol-fed Wistar rats (5.0 g/kg per 12 h for 3 times)	Quercetin 100mg/kgBW per day for 14 days	—	Antioxidation: ↑HO-1 ↑GSH-Px; ↓MDA; ↓ROS	Nrf2 NF-κB	Liu (2019)
						Antiinflammation: ↓TNF-α ↓IL-1β; ↓IL-18		
	Puerarin	Pueraria montana var. lobata (Willd.) Maesen & S.M.Almeida ex Sanjappa & Predeep [Fabaceae]	Acute alcohol-fed Wild-type AB zebrafish was incubated (2% ethanol) for 32 h	Puerarin (50 μM, 25 μM, and 12.5 μM) for 48 h	↓TG; ↓TC	Antiinflammation: ↓TNF-α ↓IL-1β	AMPK ACC	Liu et al. (2021b)
			Acute alcohol-fed C57BL/6J mice (20% EtOH 5 g/kg for one time)	Puerarin (25, 50, and 100 mg/kg) for one time	↓AST; ↓ALT	Antiinflammation: ↓TNF-α ↓IL-1β; ↓IL-6	SREBP-1c PPAR-α NF-κB	Hu et al. (2023b)
			Acute alcohol-fed AML12 cells (100 mmol/L EtOH for 24 h)					
			Acute alcohol-fed C57BL/6 mice (31.5% EtOH 5 g/kg for 10 days)	Puerarin (80 mg/kg) for 10 days	—	FAS: ↓Scd1 ↑ACOX; ↑CPT1A	NF-κB	Jingwen (2023)
	Tectoridin	Pueraria montana var. lobata (Willd.) Maesen & S.M.Almeida ex Sanjappa & Predeep [Fabaceae]	Acute alcohol-fed C57BL/6 mice (5.0 g/kg per 12 h for 3 times)	Tectoridin (25, 50, and 100 mg/kg) for one time	↓AST; ↓ALT ↓TG	Antioxidation: ↑GSH-Px ↓MDA; ↑SOD	PPAR-α	Xiong et al. (2010)
						FAS: ↑ACD ↑ACO; ↑CYP 4A		
	Genistein	Legumes, Trifolium pratense L. [Fabaceae]	Chronic alcohol-fed Wild-type (WT) C57BL/6N mice	Genistein (1 mg/kg/day) mixed in the diet for 8 weeks	↓AST; ↓ALT ↓TG; ↓TC	Antioxidation: ↓MDA ↑SOD; ↑GSH ↑HO-1; ↓ROS	Nrf2	Ding et al. (2023)
	Hydroxysafflor yellow A	Carthamus tinctorius L. [Asteraceae]	Chronic and binge alcohol-feed SPF C57BL/6 mice for 16 days	HSYA 2.5 and 7.5 mg/kg for one time	↓AST; ↓ALT ↓TG; ↓TC ↑HDL-C	Antioxidation: ↓CYP2E1 ↓MDA; ↑SOD; ↑GSH	PPAR-α; Nrf2	Wang et al. (2024)
	Baicalin	Scutellaria baicalensis Georgi [Lamiaceae]	Chronic (4 or 8 weeks) alcohol-fed (5 or 10 mL/kg/day) Wistar rats	Baicalin 120 mg/kg/day for 4 weeks	↓AST; ↓ALT; ↓TG	Antioxidation: ↑GSH-Px ↓MDA; ↑SOD	Sonic hedgehog (Shh)	Wang et al. (2016)
						Antiinflammation: ↓TNF-α ↓IL-1β; ↓IL-6		
			Chronic (6 weeks) 65% alcohol-fed C57BL/6 mice	Baicalin 50 mg/kg/day for 6 weeks	↓AST; ↓ALT ↓TG	Antioxidation: ↑GSH-Px ↓MDA; ↑SOD; ↓ROS	NF-κB	Fang et al. (2022a)
						Antiinflammation: ↓TNF-α ↓IL-1β; ↓IL-6		
	Silymarin	Silybum marianum (L.) Gaertn. [Asteraceae]	Acute alcohol-fed (5 g/kg) C57BL/6 mice per 12 h for 3 times	Silymarin 200 mg/kg mixed in alcohol	↓ALT	Antioxidation: ↑GSH	—	Song et al. (2006)
						Antiinflammation: ↓TNF-α		
			Chronic (6 weeks) 6–8 g/kg/d alcohol-fed Sprague-Dawley rats	Silymarin 100 mg/kg/d, 150 mg/kg/d, 200 mg/kg/d	↓AST; ↓ALT ↓TG	Antioxidation: ↑GSH-Px ↑SOD; ↓MDA	NF-κB	Zhang et al. (2013)
			Chronic ALD human patients and early liver fibrotic lesions	Silymarin capsule 140 mg/d for 48 weeks	↓AST; ↓ALT	Antioxidation: ↓MDA; ↑SOD	—	Kainan et al. (2018)
	Dihydromyricetin	Hovenia dulcis Thunb. [Rhamnaceae]	Chronic (5 weeks) alcohol-fed C57BL/6 mice	Dihydromyricetin 6 mg/mL for 5 weeks	↓AST; ↓ALT ↓TG; ↓TC	Antiinflammation: ↓TNF-α ↓IL-17; ↓IL-6	NF-κB	Janilkarn-Urena et al. (2023)
			Chronic (8 weeks) alcohol-fed C57Bl/6J mice	Dihydromyricetin 5 and 10 mg/kg for 6 weeks	↓AST; ↓ALT	Antiinflammation: ↓TNF-α; ↓IL-8	SREBP-1 AMPK Nrf2	Silva et al. (2020)
						Antioxidation: ↑NAD+ ↑NAD+/NADH; ↓CYP2E1 ↑HO-1; ↓ROS		
Polyphenols	Honokiol	Magnolia officinalis Rehder & E.H.Wilson [Magnoliaceae]	Chronic (6 weeks) alcohol-fed Wistar rats	Honokiol (10 mg/kg/day) for 2 weeks	↓ALT; ↓TG	Antioxidation: ↑GSH	SREBP-1c	Yin et al. (2009)
	Resveratrol	Veratrum album L. [Melanthiaceae]	Chronic (16 weeks) alcohol-fed Wistar rats	Resveratrol (250 mg/kg/day) for 16 weeks	↓AST; ↓ALT	Antioxidation: ↑SOD ↑GPx; ↑CAT; ↓CYP2E1	—	Peiyuan et al. (2017)
	Polydatin	Reynoutria japonica Houtt. [Polygonaceae]	Wild-type zebrafish exposed to 350 mM ethanol (2%EtOH) for 32 h	Polydatin 6.25, 12.5, 25 μg/mL and 0.1% for 48 h	—	Antioxidation: ↓CYP2Y3 ↓ROS	—	Lai et al. (2018)
			Chronic alcoholic human patients	Polydatin 40 mg twice a day last 2 weeks	↓AST; ↓ALT	Antioxidation: ↓MDA	—	Pace et al. (2015)
	Curcumin	Curcuma longa L. [Zingiberaceae]	Chronic (6 weeks) alcohol-fed (2.4 and 4 g/kg/day) Balb/c mice	Curcumin (75 mg/kg/day) for 6 weeks	↓AST; ↓ALT ↓TG; ↓TC	Antioxidation: ↑SOD ↑GPx; ↑GST ↓ROS; ↓MDA	NF-κB	Rong et al. (2012)
			Chronic (9 weeks) alcohol-fed Sprague-Dawley rats	Curcumin at 100, 200, and 400 mg/kg for 9 weeks	↓AST; ↓ALT ↓TG; ↓TC	Antiinflammation: ↓TNF-α	NF-κB SREBP-1c PPAR-α Nrf2	Lu et al. (2015)
			Chronic (4 weeks) alcohol-fed Wistar rats	Curcumin (75 mg/kg/day) for 4 weeks	↓ALT	Antiinflammation: ↓TNF-α ↓IL-12; ↓MCP-1	NF-κB	Nanji et al. (2003)
			Chronic (4 weeks) alcohol-fed Kunming mice	Curcumin 50 mg/kg/d, 100 mg/kg/d, 200 mg/kg/d for 4 weeks	↓AST; ↓ALT ↓TG	Antioxidation: ↑GSH ↑SOD; ↓MDA	—	Dong and Xu (2017)
						Antiinflammation: ↓TNF-α ↓MCP-1		
	Salvianolic acid	Salvia miltiorrhiza Bunge [Lamiaceae]	Chronic (8 weeks) alcohol-fed Sprague-Dawley rats	Salvianolic acid A (8 and 16 mg/kg/day) for 8 weeks	↓AST; ↓ALT ↓TG; ↓TC	Antioxidation: ↑GSH ↓MDA; ↑SOD-l ↓CYP2E1	SIRT1	Shi et al. (2018)
			Chronic (8 weeks) alcohol-fed C57BL/6 mice	Salvianolic acid A (20 mg/kg/day) for 7 weeks	↓ALT; ↓TG	—	PPAR-α	Zhang (2024)
			Chronic (8 weeks) alcohol-fed Sprague-Dawley rats	Salvianolic acid B (15 and 30 mg/kg/day) for 8 weeks	↓AST; ↓ALT	Antioxidation: ↓CYP2E1	SIRT1	Zhang et al. (2017)
						Antiinflammation: ↓TNF-α; ↓IL-6		
			Acute alcohol-fed (6 g/kg every 12 h for 3 times) Wistar rats	Salvianolic acid B (15 and 30 mg/kg/day) for 3 days	↓AST; ↓ALT	Antiinflammation: ↓TNF-α; ↓IL-6	NF-κB SIRT1	Li et al. (2014)
						Antioxidation: ↑GSH ↑GSH-PX; ↓MDA		
			Chronic (8 weeks) alcohol-fed Sprague-Dawley rats	Salvianolic acid B (15 and 30 mg/kg/day) for 8 weeks	↓AST; ↓ALT ↓TG; ↓TC	—	SIRT1 AMPK	Qiu et al. (2020)
Terpenoids	Oleanolic acid	Olea europaea L. [Oleaceae], Ligustrum lucidum W.T.Aiton [Oleaceae]	Chronic (30 days) alcohol-fed Sprague-Dawley rats	Oleanolic acid (10 mg/kg/day) for 30 days	↓AST; ↓ALT ↓TG; ↓TC	Antioxidation: ↑GSH ↓MDA; ↓GSSG; ↑HO-1 ↑SOD-l; ↑GR; ↓CYP2E1	Nrf2	Liu et al. (2014)
						Antiinflammation: ↓TNF-α↓IL-6		
	Ursolic acid	Arctostaphylos uva-ursi (L.) Spreng. [Ericaceae]	Chronic ALD human patients	3 ursolic acid extended-release tablets per day last 28 days	↓AST; ↓ALT	Antioxidation: ↓MDA; ↑SOD	—	Zhao et al. (2019)
			1 week alcohol-fed Kunming mice	20, 40, or 80 mg/kg/d Ursolic acid for 1 week	↓AST; ↓ALT	Antioxidation: ↑SOD	CASP3	Ma et al. (2021)
	Tanshinones	Salvia miltiorrhiza Bunge [Lamiaceae]	Chronic (4 weeks) alcohol-fed C57BL/6 mice	20 and 40 mg/kg/d Cryptotanshinone for 4 weeks	↓AST; ↓TG	Antioxidation: ↓MDA ↑CAT; ↑SOD ↑GSH; ↑GPx	SREBP-1c AMPK/SIRT1 Nrf2 NF-κB	Nagappan et al. (2019)
Alkaloids	Oxymatrine	Sophora flavescens Aiton [Fabaceae]	Acute once alcohol-fed (50% 12 mL/kgd) Kunming mice	1.4 and 2.8 mg/kg/d Oxymatrine for 2 weeks	↓AST; ↓ALT ↓TG	Antioxidation: ↓MDA ↑SOD; ↑CAT; ↑GSH	—	Du et al. (2021)
			Chronic (8 weeks) alcohol-fed (40% 15 mL/kg/d) Sprague-Dawley rats	22.22, 44.44 and 66.66 mg/kg/d Oxymatrine for 6 weeks	↓AST; ↓ALT	Antiinflammation: ↓TNF-α ↓IL-1β; ↓IL-6	MAPK/NF-κB	Huang et al. (2023)
	Berberine	Coptis chinensis Franch. [Ranunculaceae], Phellodendron chinense C.K.Schneid. [Rutaceae]	Acute (6 g/kg for 3 times) alcohol-fed ICR mice	Berberine 200 and 300 mg/kg/day for 10 days	↓ALT	Antioxidation: ↑GSH	—	Zhang et al. (2014)
			Chronic (36% caloric content for 5 weeks) alcohol-fed ICR mice	Berberine 120 mg/kg/day for 5 weeks	↓ALT; ↓TG ↓TC	Antioxidation: ↓CYP2E1	PPAR-α/PGC-1α	
Saponins	Dioscin	Dioscorea nipponica Makino [Dioscoreaceae], Polygonum aviculare L. [Polygonaceae]	Acute alcohol-fed (4 days) C57BL/6 mice	Dioscin 28, 56 and 84 mg/kg/d	↓AST; ↓ALT ↓TG; ↓TC	Antioxidation: ↓MDA ↑GSH-Px; ↑SOD; ↑GSH ↑GSR; ↓CYP2E1	MAPK/NF-κB PPAR-α	Xu et al. (2014)
						Antiinflammation: ↓TNF-α; ↓IL-6		
			Chronic (8 weeks) alcohol-fed Wistar rats	Dioscin 20, 40 and 60 mg/kg/d		Antioxidation: ↓MDA ↑GSH-Px; ↑SOD; ↑GSH ↑GSR; ↓CYP2E1		
						Antiinflammation: ↓TNF-α; ↓IL-6		
	Saponins of Panax japonicus	Panax japonicus (T.Nees) C.A.Mey. [Araliaceae]	Chronic (30 days) alcohol-fed (56% 14.2 mL/kg) ICR mice	Saponin of Panax japonicus 12.5, 25 and 50 mg/kg for 30 days	↓AST; ↓ALT	Antioxidation: ↓MDA ↑SOD; ↑GSH ↑GPX; ↑CAT	—	Li et al. (2010)
			Acute (3 days) alcohol-fed (50% 5 g/kg) C57BL/6 mice	Saponin of Panax japonicus 75 and 150 mg/kg/d	↓AST; ↓ALT ↓TG	Antioxidation: ↓MDA ↑SOD; ↑GSH ↑CAT; ↓CYP2E1	Nrf2 AMPK-ACC/PPAR-α	Qiu et al. (2023)
	Betulinic acid	Syzygium cumini (L.) Skeels [Myrtaceae], Betula pendula Roth [Betulaceae], Ziziphus jujuba Mill. [Rhamnaceae]	Acute (once) alcohol-fed (50% 10 mL/kg) Kunming mice	Betulinic acid 0.25, 0.5, and 1.0 mg/kg/d for 14 days	↓AST; ↓ALT ↓TG; ↓TC	Antioxidation: ↓MDA ↑GSH-Px; ↑SOD; ↑CAT	—	Yi et al. (2014)
	Ginsenoside Rg1	Panax ginseng C.A.Mey. [Araliaceae], Panax notoginseng (Burkill) F.H.Chen [Araliaceae]	10 days alcohol-fed C57BL/6 mice	Ginsenoside Rg1 40 mg/kg twice per day for 2 days	↓AST; ↓ALT	Antioxidation: ↓CYP2E1 ↓ROS; ↓MDA; ↑GSH-Px	—	Yang et al. (2021a)
Anthraquinones	Purpurin	Rubia tinctorum L. [Rubiaceae]	3 weeks alcohol-fed Swiss albino mice	Purpurin 40 and 80 mg/kg/d for 3 weeks	—	Antioxidation: ↓ROS ↓CYP2E1; ↑SOD; ↑GST ↑CAT; ↑GPx; ↓MDA; ↑GSH	Nrf2	Hussain et al. (2022)
	Aloin	Aloe vera (L.) Burm.f. [Asphodelaceae]	Chronic (11 weeks) alcohol-fed Kunming mice	Aloin 10 and 30 mg/kg/d for 11 weeks	↓AST; ↓ALT ↓TG; ↓TC	Antioxidation: ↓MDA ↑SOD; ↑CAT; ↓CYP2E1	SREBP-1c AMPK	Cui et al. (2014)
						Antiinflammation: ↓TNF-α; ↓IL-10		

In the table, “mg/kg/d” represents the oral dose of the metabolite in mice per day; concentration units (μM, μg/mL) represent the concentration of the metabolite in the drinking water or in the cell treatment. Sterol regulatory element-binding proteins (SREBPs), peroxisome proliferator-activated receptor-alpha (PPAR-α), AMP-activated protein kinase (AMPK), mitogen-activated protein kinase (MAPK), nuclear factor erythroid 2-related factor 2 (Nrf2), sirtuins1 (SIRT1), and nuclear factor kappa B (NF-κB), fatty acid synthase (FAS), glutathione (GSH), oxidized glutathione (GSSG), 4-hydroxynonenal (4-HNE) and malondialdehyde-acetaldehyde adducts (MAA); superoxide dismutase (SOD), catalase (CAT), glutathione peroxidase (GPx), glutathione reductase (GR), glutathione S-transferase (GST), γ-glutamyltransferase (γ-GT), carnitine palmitoyltransferase 1 (CPT-1), lactate dehydrogenase (LDH), heme oxygenase-1 (HO-1), xanthine oxidase (XO); tumor necrosis factor-alpha (TNF-α) and interleukins-1β/interleukins-6 (IL-1β/IL-6).

### 2.1 Inclusion criteria

(1) The study design should be conducted in rats, mice, or clinical patients; (2) The experimental design must include ALD (induced by alcohol/ethanol at any dose and duration); (3) The study must involve Chinese botanical drugs; (4) The study must include a certain dosage of antioxidant enzymes (e.g., SOD, catalase, glutathione peroxidase, glutathione reductase, etc.) and a certain dosage of oxidative damage markers [e.g., malondialdehyde (MDA), etc.]; (5) A control group should be included for comparison with the ALD group.

### 2.2 Exclusion criteria

(1) Animals or patients with comorbidities; (2) Animals with ALD; (3) *In vitro* or cell culture studies; (4) Botanical drugs other than traditional Chinese botanical drugs; (5) Studies lacking oxidative stress biomarkers; (6) Control animals exposed to substances other than water, phosphate-buffered saline (PBS), methylcellulose, or inert substances; (7) Studies without a separate control group; (8) Case studies, crossover studies, abstracts, case reports, letters to the editor, editorials, or reviews; (9) Computer simulations; (10) Studies with flawed experimental design and incomplete data reporting.

### 2.3 Search strategy

A literature search using both Chinese and English keywords was conducted in the Web of Science, PubMed, and CNKI databases, up to 24 December 2024. The following keywords were used in the search strategy: ((((((((((((((((((((((((((Quercetin [Title/Abstract]) OR (Puerarin [Title/Abstract])) OR (Tectoridin [Title/Abstract])) OR (Genistein [Title/Abstract])) OR (Hydroxysafflor Yellow A [Title/Abstract])) OR (Baicalin [Title/Abstract])) OR (Silymarin [Title/Abstract])) OR (Dihydromyricetin [Title/Abstract])) OR (Honokiol [Title/Abstract])) OR (Resveratrol [Title/Abstract])) OR (Polydatin [Title/Abstract])) OR (Curcumin [Title/Abstract])) OR (Salvianolic Acid [Title/Abstract])) OR (Oleanolic Acid [Title/Abstract])) OR (Ursolic Acid [Title/Abstract])) OR (Tanshinones [Title/Abstract])) OR (Oxymatrine [Title/Abstract])) OR (Berberine [Title/Abstract])) OR (Dioscin [Title/Abstract])) OR (Saponins of Panax japonicus [Title/Abstract])) OR (Betulinic Acid [Title/Abstract])) OR (Ginsenoside Rg1 [Title/Abstract])) OR (Purpurin [Title/Abstract])) OR (Aloin [Title/Abstract])) OR ((((((((((((((((((((((((((((((((((((((((((((((((((Drugs, Chinese Herbal [Title/Abstract]) OR (Chinese Drugs, Plant [Title/Abstract])) OR (Chinese Herbal Drugs [Title/Abstract])) OR (Herbal Drugs, Chinese [Title/Abstract])) OR (Plant Extracts, Chinese [Title/Abstract])) OR (Chinese Plant Extracts [Title/Abstract])) OR (Extracts, Chinese Plant [Title/Abstract])) OR (Flavonoids [Title/Abstract])) OR (2-Phenyl-Chromene [Title/Abstract])) OR (2 Phenyl Chromene [Title/Abstract])) OR (Flavonoid [Title/Abstract])) OR (2-Phenyl-Benzopyran [Title/Abstract])) OR (2 Phenyl Benzopyran [Title/Abstract])) OR (2-Phenyl-Benzopyrans [Title/Abstract])) OR (2 Phenyl Benzopyrans [Title/Abstract])) OR (2-Phenyl-Chromenes [Title/Abstract])) OR (2 Phenyl Chromenes [Title/Abstract])) OR (Bioflavonoids [Title/Abstract])) OR (Bioflavonoid [Title/Abstract])) OR (Polyphenols [Title/Abstract])) OR (Polyphenol [Title/Abstract])) OR (Provinols [Title/Abstract])) OR (Terpenes [Title/Abstract])) OR (Terpenoid [Title/Abstract])) OR (Terpene [Title/Abstract])) OR (Terpenoids [Title/Abstract])) OR (Isoprenoids [Title/Abstract])) OR (Isoprenoid [Title/Abstract])) OR (Alkaloids [Title/Abstract])) OR (Alkaloid [Title/Abstract])) OR (Plant Alkaloids [Title/Abstract])) OR (Alkaloids, Plant [Title/Abstract])) OR (Plant Alkaloid [Title/Abstract])) OR (Alkaloid, Plant [Title/Abstract])) OR (Saponins [Title/Abstract])) OR (Saponin [Title/Abstract])) OR (Anthraquinones [Title/Abstract])) OR (Anthracenedione [Title/Abstract])) OR (Anthranoid [Title/Abstract])) OR (Anthraquinone Compound [Title/Abstract])) OR (Compound, Anthraquinone [Title/Abstract])) OR (Anthraquinone Derivative [Title/Abstract])) OR (Derivative, Anthraquinone [Title/Abstract])) OR (Anthraquinone [Title/Abstract])) OR (Anthracenediones [Title/Abstract])) OR (Anthranoids [Title/Abstract])) OR (Anthraquinone Compounds [Title/Abstract])) OR (Anthraquinone Derivatives [Title/Abstract])) OR (Dianthrones [Title/Abstract])) OR (Dianthraquinones [Title/Abstract]))) AND (((((((((((((((((((((((((((((((((((((((((Oxidative Stress [Title/Abstract]) OR (Oxidative Stresses [Title/Abstract])) OR (Stress, Oxidative [Title/Abstract])) OR (Oxidative DNA Damage [Title/Abstract])) OR (Damage, Oxidative DNA [Title/Abstract])) OR (DNA Damage, Oxidative [Title/Abstract])) OR (Oxidative DNA Damages [Title/Abstract])) OR (DNA Oxidative Damage [Title/Abstract])) OR (Damage, DNA Oxidative [Title/Abstract])) OR (DNA Oxidative Damages [Title/Abstract])) OR (Oxidative Damage, DNA [Title/Abstract])) OR (Oxidative [Title/Abstract] AND Nitrosative Stress [Title/Abstract])) OR (Nitro-Oxidative Stress [Title/Abstract])) OR (Nitro Oxidative Stress [Title/Abstract])) OR (Nitro-Oxidative Stresses [Title/Abstract])) OR (Stresses, Nitro-Oxidative [Title/Abstract])) OR (Stress, Nitro-Oxidative [Title/Abstract])) OR (Oxidative Nitrative Stress [Title/Abstract])) OR (Nitrative Stress, Oxidative [Title/Abstract])) OR (Oxidative Nitrative Stresses [Title/Abstract])) OR (Stress, Oxidative Nitrative [Title/Abstract])) OR (Oxidative Damage [Title/Abstract])) OR (Damage, Oxidative [Title/Abstract])) OR (Oxidative Damages [Title/Abstract])) OR (Oxidative Injury [Title/Abstract])) OR (Injury, Oxidative [Title/Abstract])) OR (Oxidative Injuries [Title/Abstract])) OR (Oxidative Stress Injury [Title/Abstract])) OR (Injury, Oxidative Stress [Title/Abstract])) OR (Oxidative Stress Injuries [Title/Abstract])) OR (Stress Injury, Oxidative [Title/Abstract])) OR (Oxidative Cleavage [Title/Abstract])) OR (Cleavage, Oxidative [Title/Abstract])) OR (Oxidative Cleavages [Title/Abstract])) OR (Antioxidative Stress [Title/Abstract])) OR (Antioxidative Stresses [Title/Abstract])) OR (Stress, Antioxidative [Title/Abstract])) OR (Anti-oxidative Stress [Title/Abstract])) OR (Anti oxidative Stress [Title/Abstract])) OR (Anti-oxidative Stresses [Title/Abstract])) OR (Stress, Anti-oxidative [Title/Abstract]))) AND (((((((((Alcohol-Related Disorder [Title/Abstract]) OR (Alcohol-Related Disorders [Title/Abstract])) OR (Alcohol Related Disorders [Title/Abstract])) OR (Disorder, Alcohol-Related [Title/Abstract])) OR (Alcohol Problem [Title/Abstract])) OR (Alcohol Problems [Title/Abstract])) OR (ALD [Title/Abstract])) OR (alcohol-related liver disease [Title/Abstract])) OR (alcoholic liver disease [Title/Abstract])). The search was restricted by English.

In addition to the above search content, we also searched the literature on the improvement of oxidative stress by exercise to explore the mechanism of improvement similar to that of Chinese herbal medicine. The search method is similar to the above.

## 3 Ethanol metabolism and oxidative stress in ALD

### 3.1 Ethanol metabolism in ALD

In a healthy liver, ethanol is primarily metabolized through three main pathways:

(1) The Alcohol Dehydrogenase (ADH) System: ADH, an oxidized nicotinamide adenine dinucleotide (NAD+)-dependent enzyme (Masia et al., 2018), converts NAD + to reduced nicotinamide adenine dinucleotide (NADH) while oxidizing ethanol to acetaldehyde (Michalak et al., 2021). Acetaldehyde, a reactive metabolite, forms adducts with cellular proteins, nucleic acids, and lipids, disrupting cellular functions (Rocco, 2014). It is rapidly converted to acetate by aldehyde dehydrogenase (ALDH) within the mitochondria and cytosol (LeFort et al., 2024). Acetate then enters the tricarboxylic acid cycle via acetyl-CoA, where it is either converted to energy in the form of ATP or broken down into carbon dioxide and water.
(2) The Microsomal Ethanol Oxidizing System (MEOS): This pathway involves the metabolism of ethanol to acetaldehyde in the mitochondria, converting NADPH and O_2_ into oxidized nicotinamide adenine dinucleotide phosphate (NADP+) and H_2_O, while simultaneously producing ROS (Lu and Cederbaum, 2008). Cytochrome P450 2E1 (CYP2E1), located in the endoplasmic reticulum and reliant on NADPH (Doody et al., 2017), plays a key role in this process. Chronic alcohol consumption induces CYP2E1 expression, enhancing ethanol metabolism in the mitochondria and increasing ROS generation, including hydrogen peroxide (H_2_O_2_) and hydroxyl radicals (OH−) (Albano, 2006).
(3) Other Oxidative Pathways: These include reduced nicotinamide adenine dinucleotide phosphate (NADPH) oxidase-catalase (CAT) and xanthine oxidase-catalase (XO-CAT) systems (Zima et al., 2001). Additionally, non-oxidative pathways regulated by fatty acid ethyl ester synthase and phospholipase D are involved in the generation of fatty acid ethyl esters and phosphatidylethanol (Park et al., 2022b).

However, chronic excessive alcohol consumption leads to significant metabolic disruptions:

(1) Disruption of the NADH/NAD + Ratio: This imbalance enhances respiratory chain activity, increases oxygen consumption, and promotes ROS formation (Yang et al., 2022). Elevated NADH inhibits mitochondrial β-oxidation and suppresses fatty acid synthesis, resulting in intracellular lipid accumulation (You and Arteel, 2019; Subramaiyam, 2023). During inflammatory processes, triglycerides undergo hydrolysis, forming free fatty acids (FFA) (Zhu et al., 2022), which are toxic to hepatocytes and induce apoptosis through both extrinsic and intrinsic pathways (LeFort et al., 2024).
(2) Toxic Lipid Metabolites: The metabolism of lipids produces toxic metabolites such as malondialdehyde (MDA), 4-hydroxynonenal (4-HNE), and malondialdehyde-acetaldehyde adducts (MAA), which further damage mitochondria and promote free radical production (Schaffert, 2009). These metabolites also trigger immune responses by interacting with proteome arrays recognized by IgG antibodies, resulting in elevated antibody levels against protein adducts, correlating with ALD severity (Ahmadi et al., 2023).

### 3.2 Oxidative stress and its role in ALD

Reactive oxygen species (ROS) are crucial regulators of cell signaling and stress responses (Nagy et al., 2016). Under normal conditions, the body maintains a delicate balance between oxidative and antioxidant systems to preserve homeostasis. However, excessive alcohol consumption disrupts this balance by generating an overabundance of ROS and other reactive molecules while impairing the body’s antioxidant defenses. This imbalance leads to oxidative stress (OS), which is a key contributor to the pathogenesis of ALD (Park et al., 2022a). Accumulating evidence indicates that OS not only triggers inflammatory responses but also directly contributes to liver damage in ALD (Louvet and Mathurin, 2015). Understanding OS is crucial for comprehending its role in ALD progression, as it indicates the body’s reduced capacity to combat the excessive generation of reactive oxygen or nitrogen species, coupled with weakened antioxidant defenses (Dukić et al., 2023).

Mitochondria, as the primary source of ROS in cells, are particularly vulnerable to OS because they have low levels of antioxidants such as glutathione (GSH) (Koch et al., 2004). GSH acts as a direct scavenger of ROS (Galligan et al., 2012). Chronic alcohol consumption impairs GSH transport proteins, causing a progressive depletion of mitochondrial GSH. This reduction makes mitochondria more susceptible to damage from free radicals, leading to mitochondrial DNA depletion and dysfunction (Salete-Granado et al., 2023).

**FIGURE 1:**
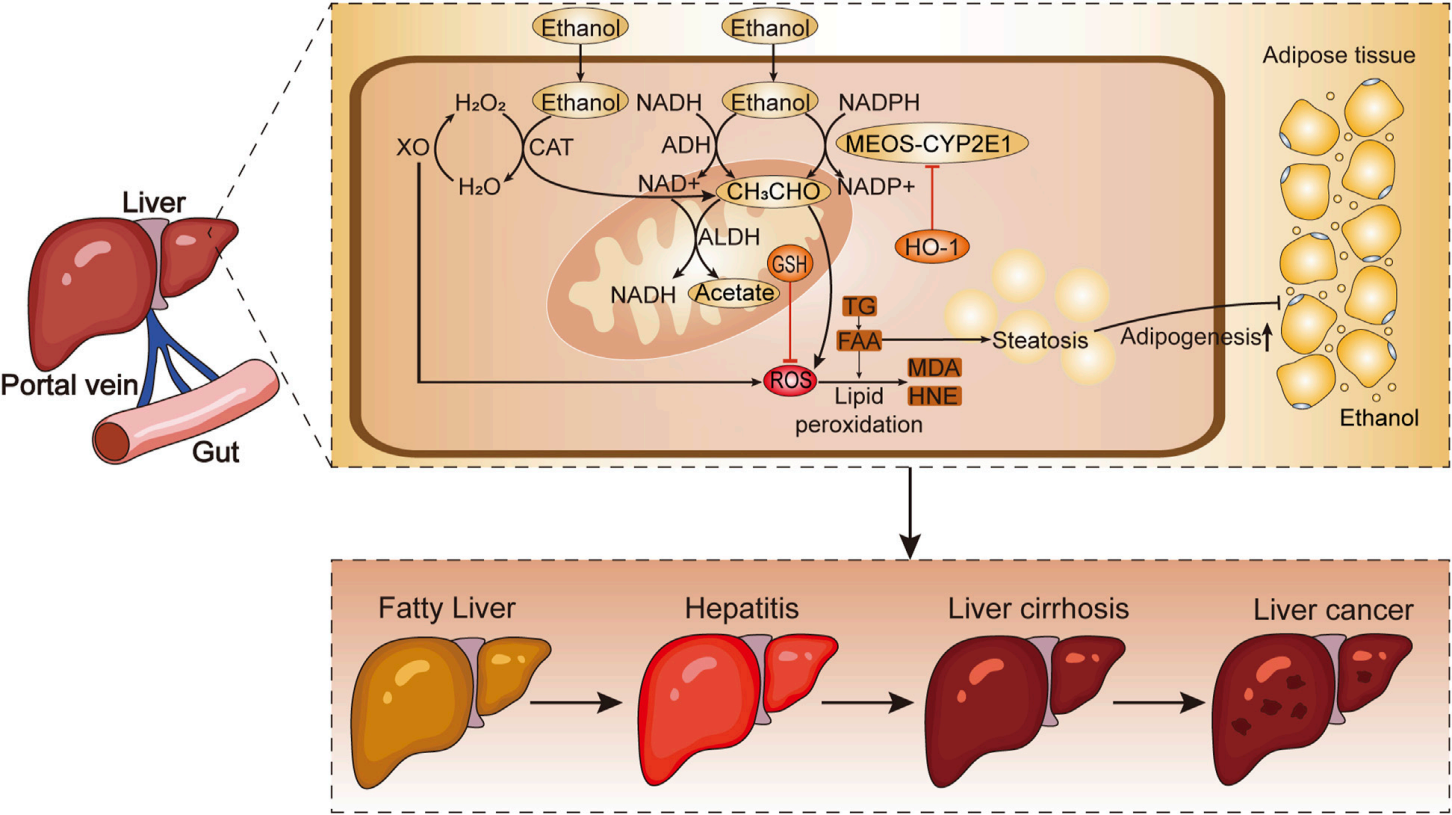
The metabolic process of alcohol absorption from the intestine into liver cells. Chronic excessive drinking leads to the production of ROS and lipid peroxides, which contribute to the development of ALD. H_2_O_2_, hydrogen peroxide; H_2_O, water; XO, xanthine oxidase; CAT, catalase; NADH, reduced nicotinamide adenine dinucleotide; NADPH, reduced nicotinamide adenine dinucleotide phosphate; NAD+, oxidized nicotinamide adenine dinucleotide; ALDH, aldehyde dehydrogenase; NADP+, oxidized nicotinamide adenine dinucleotide phosphate; HO-1, heme oxygenase-1; CH_3_CHO, acetaldehyde; GSH, glutathione; ROS, reactive oxygen species; TG, triglycerides; FAA, free fatty acids; MDA, malondialdehyde; HNE, 4-hydroxynonenal; MEOS-CYP2E1, microsomal ethanol ixidizing system-cytochrome P450 2E1.

Collectively, these findings suggest that ROS-induced OS plays a pivotal role in steatosis, inflammation, and apoptosis, contributing significantly to the progression of ALD. The disturbances in glucose and lipid metabolism associated with OS further exacerbate the condition (Seth et al., 2011) (Figure 1).

## 4 Biological measurement indicators

A variety of biological parameters are employed to assess OS in ALD (Wu and Cederbaum, 2009). These indicators (Moreno et al., 2019) include cellular or serum markers such as total cholesterol (TC), glutamic oxaloacetic transaminase (AST); triglycerides (TG), glutamic pyruvic transaminase (ALT), 4-hydroxynonenal (4-HNE); malondialdehyde-acetaldehyde adducts (MAA) and nitrogen oxides. In addition, antioxidant enzymes are used to evaluate OS (Grasselli et al., 2014), including cytochrome P450 2E1 (CYP2E1); lactate dehydrogenase (LDH), superoxide dismutase (SOD), heme oxygenase-1 (HO-1), glutathione peroxidase (GPx), carnitine palmitoyltransferase 1 (CPT-1), catalase (CAT), xanthine oxidase (XO), oxidized glutathione (GSSG), glutathione S-transferase (GST), glutathione reductase (GR), protein kinase C, oxidoreductase, and lipoxygenase. Corresponding reagent kits or test kits (such as for ALT, AST, etc.) are commonly used, and biochemical analysis (such as enzyme-linked immunosorbent assay) is performed using appropriate instruments (such as a microplate reader, etc.).

Additionally, gene expression patterns like oxidized DNA and cellular conditions, such as cell viability, hemolysis, and apoptosis, can help clarify the extent of OS (Liu et al., 2023). Cytokines and inflammatory markers, including tumor necrosis factor-alpha (TNF-α) and interleukins 1β or 6 (IL-1β, IL-6), further indicate the level of hepatocyte-macrophage apoptosis (Hu et al., 2025).

## 5 Signaling pathways/cytokines of ALD oxidative stress

**FIGURE 2:**
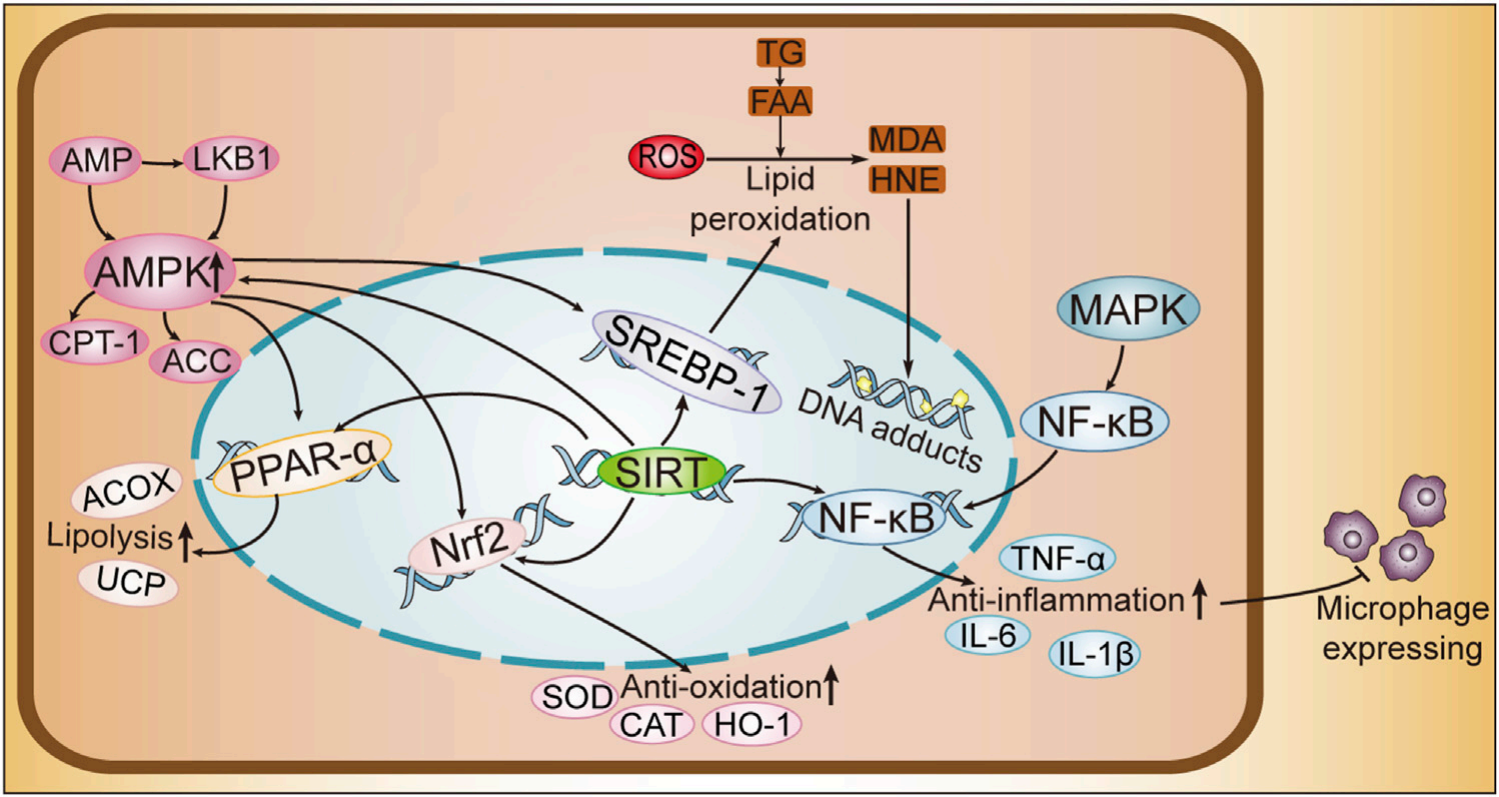
Cellular Pathway Mechanism Diagram of ALD. SREBPs, sterol regulatory element-binding proteins; PPAR-α, peroxisome proliferator-activated receptor-alpha; AMPK, AMP-activated protein kinase; MAPK, mitogen-activated protein kinase; Nrf2, nuclear factor erythroid 2-related factor 2; SIRT1, sirtuins1; NF-κB, nuclear factor kappa B; AMP, adenosine monophosphate; LKB1, liver kinase B1; CPT-1, carnitine palmitoyltransferase 1; ACC, acetyl-CoA carboxylase; ACOX, acyl-CoA oxidase; UCP, uncoupling protein; SOD, superoxide dismutase; CAT, catalase; HO-1, heme oxygenase-1; IL-6, interleukin-6; IL-1β, interleukin-1 beta; TNF-α, tumor necrosis factor alpha; ROS, reactive oxygen species; TG, triglycerides; FFA, free fatty acids; MDA, malondialdehyde; HNE, 4-hydroxynonenal.

Multiple signaling pathways implicated to the pathogenesis of ALD, including. These pathways are potential therapeutic targets for ALD and play pivotal roles in its treatment (Figure 2).

Sterol regulatory element-binding proteins (SREBPs) are transcription factors that modulate the biosynthetic pathways of cholesterol, fatty acids, and triglycerides within hepatic and extrahepatic tissues. These proteins exist in three subtypes: SREBP-1a, SREBP-1c, and SREBP-2, each contributing to distinct metabolic pathways (Li et al., 2011). SREBP-1 predominantly governs fatty acid biosynthesis, whereas SREBP-2 is primarily implicated in cholesterol biosynthesis. These proteins are initially synthesized as precursors that associate with the endoplasmic reticulum and nuclear membranes, with SREBP-1c representing the most abundant isoform in hepatic tissue (Ceni et al., 2014). Alcohol and its metabolite, acetaldehyde, promote SREBP-1 protein synthesis, thereby increasing hepatic steatosis and contributing to ALD progression (Gao and Bataller, 2011).

Peroxisome proliferator-activated receptor-alpha (PPAR-α) serves as a pivotal transcription factor that orchestrates the transcriptional regulation of genes containing peroxisome proliferator response elements (PPREs) (Yamazaki et al., 2025). These genes are involved in fatty acid oxidation, transport, and export (Pi et al., 2021). Additionally, PPAR-α influences the activity of mitochondrial uncoupling proteins (UCP) and acyl-CoA oxidase 1 (ACOX), both of which regulate ROS production. Ethanol reduces PPAR-α activity, resulting in the accumulation of fatty acids in liver cells (Meng et al., 2020).

Chronic alcohol consumption disrupts hepatic lipid metabolism through the SREBP and PPAR-α pathways, leading to steatosis. These two receptors are directly modulated by AMP-activated protein kinase (AMPK), an enzyme integral to lipid metabolism, which exerts its influence through phosphorylation mechanisms (Hsu et al., 2014). AMPK regulates energy homeostasis by inhibiting anabolic pathways (thus preventing further ATP consumption) and promoting catabolic pathways (thereby increasing ATP production), ultimately reducing OS. AMPK also activates fatty acid oxidation and inhibits lipogenesis (Attal et al., 2022). However, chronic ethanol consumption inhibits AMPK activity by blocking the phosphorylation of enzymes essential for lipid metabolism, such as acetyl-CoA carboxylase (ACC) and CPT-1, resulting in reduced fatty acid oxidation (Hwang et al., 2025).

Mitogen-activated protein kinases (MAPKs) constitute a distinct family of serine-threonine protein kinases, which are activated by a variety of extracellular signals (Han, 2016). They are key regulators of cell proliferation, differentiation, apoptosis, and stress responses. In the liver, MAPK regulates lipid metabolism by phosphorylating target enzymes, promoting fatty acid oxidation through the inactivation of ACC and the activation of malonyl-CoA decarboxylase (MCD) (Zhao et al., 2022a). ACC facilitates the conversion of acetyl-CoA to malonyl-CoA, which serves as a precursor for fatty acid synthesis. Through the inhibition of ACC and the activation of MCD, MAPK diminishes malonyl-CoA levels, leading to a reduction in fatty acid synthesis and an increase in fatty acid catabolism (Fang et al., 2022a). Additionally, MAPK has the capacity to modulate SREBP-1 activation and influence mitochondrial fatty acid oxidation, potentially advancing the progression of alcoholic fatty liver disease (AFLD) (Bi et al., 2015).

Nuclear factor erythroid 2-related factor 2 (Nrf2) is a critical transcription factor in cellular antioxidant responses (Sul and Ra, 2021). Acetaldehyde interferes with glutathione activity, which reduces Nrf2 expression. Nrf2 interacts with antioxidant response elements (ARE) situated within the regulatory regions of genes encoding antioxidant enzymes, thereby activating OS (Zhao et al., 2018). In ALD, Nrf2 deficiency enhances lipogenesis by promoting SREBP-1c activity, while also increasing β-oxidation. When Nrf2 responses are inadequate, lipid peroxides can trigger inflammation through the nuclear factor kappa B (NF-κB) pathway (Liu et al., 2021a).

NF-κB functions as a principal modulator of inflammatory processes (Kong et al., 2021), operating as a nuclear transcription factor that orchestrates the transcription of a diverse array of inflammatory genes, including TNF-α (Nowak and Relja, 2020). In Kupffer cells (Louvet et al., 2011), Nrf2 and NF-κB have opposing roles in regulating inflammation (Chen et al., 2022). While NF-κB promotes inflammation, Nrf2 alleviates these effects through the inhibition of the NF-κB signaling pathway (Lu et al., 2016).

Sirtuins, particularly SIRT1(Zhao et al., 2022b), are NAD-dependent deacetylases that regulate enzymes involved in fatty acid catabolism, such as AMPK, PPAR-α, SREBP-1c, and Nrf2. By regulating these pathways, SIRT1 reduces ethanol-induced hepatic lipid accumulation and OS (Ren et al., 2020). Additionally, SIRT1 inhibits alcohol-mediated inflammation through the NF-κB pathway and decreases the expression of fibroblast markers, thereby alleviating liver fibrosis (Liu et al., 2022).

## 6 Current treatments in ALD

Abstinence from alcohol is the most crucial treatment for patients with alcoholic liver disease (ALD) at any stage (Dukewich et al., 2025). In its early stages, ALD manifests as fatty liver and mild hepatitis; abstinence can reduce inflammation in these patients. Clinically, alcohol withdrawal medications can serve as an auxiliary treatment to support abstinence. After long-term abstinence, the course of ALD can be partially reversed. Alcohol withdrawal syndrome (AWS), a side effect of quitting drinking, requires additional pharmacological symptomatic treatment (Reyes-Fermin et al., 2020). Patients with early-stage ALD often suffer from malnutrition or infections due to protein damage, so nutritional therapy should be implemented, focusing on providing high-protein, low-fat diets while balancing vitamin and folic acid levels within the body (Gao and Bataller, 2011).

As ALD progresses to severe alcoholic hepatitis and cirrhosis, treatment guidelines recommend adding hormonal therapy to the early-stage treatments to prevent and halt the progression of cirrhosis. When ALD advances to cirrhosis, liver failure, or hepatocellular carcinoma, where the liver loses normal function and conventional treatments are ineffective, liver transplantation becomes the most effective method to increase survival rates by providing a normally functioning liver. Patients must achieve long-term abstinence to qualify for liver transplantation, underscoring the importance of abstinence, although the shortage of liver donors remains a significant challenge (Singh et al., 2017).

Recent studies have found that mesenchymal stem cell therapy may be a promising approach for cirrhotic patients. Mesenchymal stem cells can suppress inflammatory responses, decrease hepatocyte apoptosis, enhance hepatocyte regeneration, reverse fibrosis, and improve liver metabolism. However, more mature research is needed to provide safe and controllable stem cell therapies (Liu et al., 2021a). Targeted therapies have also been explored in recent years, intervening in specific cellular pathways and targets to modulate disease progression (Table 1).

Moreover, numerous studies indicate that various natural metabolites can help treat ALD. Chinese herb medicine and botanical drugs are increasingly gaining attention as alternative treatments for ALD. Clinical evidence shows that botanical drugs can inhibit steatosis and inflammation progression in alcoholic liver disease. The Chinese Clinical Registration Platform has documented that Jiuganqing capsules, made from botanical drugs like flavonoids and saponins, are already in Phase II a clinical trials, and Yigan Mingmu Oral Liquid, which contains multiple Chinese herbal components, has been launched on the market. Mechanisms related to preventing hepatic steatosis and halting the deterioration of hepatitis have been demonstrated (Li et al., 2015). Biologically, botanical drugs can lower serum AST, ALT, TG, TC levels, reduce lipogenesis, enhance β-oxidation, decrease ROS, MDA, CYP2E1, and GSSG levels, increase SOD, GST, CAT, GPx, GSH, HO-1, GR levels, thereby achieving antioxidant and anti-oxidative stress effects (Wang et al., 2016). They can also lower TNF-α, IL-1β, IL-6 levels, achieving anti-inflammatory effects by inhibiting the activation of inflammatory pathways. Recent studies have highlighted key molecular targets such as SREBP (Attal et al., 2022), PPAR-α (Yue et al., 2021), AMPK (Fang et al., 2022a), MAPK (Huang et al., 2023), Nrf2 (Lu et al., 2016), NF-κB (Kong et al., 2021), and SIRT (Ren et al., 2020) as mechanisms by which Chinese botanical drugs regulate lipid metabolism in the liver.

Exercise, as a non-pharmacological intervention, has demonstrated significant potential in the treatment of ALD. Research indicates that both aerobic exercise (Huang et al., 2024) and high-intensity interval training (HIIT) (Fredrickson et al., 2021) markedly enhance liver metabolism via activation of crucial pathways including NF-κB, AMPK, and Nrf2. On the one hand, these interventions enhance fatty acid oxidation and suppress lipid synthesis, mitigating fat buildup in the liver. On the other hand, they reduce levels of pro-inflammatory cytokines such as TNF-α and IL-6, as well as oxidative stress markers like MDA. They also upregulate antioxidant enzyme activity (Bekheit et al., 2025). These mechanisms of action overlap significantly with the therapeutic targets of phytomedicines. This suggests that combining exercise interventions with phytomedicine may produce synergistic therapeutic effects.

## 7 Effect of Chinese botanical drugs on oxidative stress of alcoholic liver disease

Numerous studies have demonstrated that traditional Chinese medicine, as a natural antioxidant, can effectively prevent or alleviate ALD (Li et al., 2015). This article reviews the origins and mechanisms of ALD, along with the preventive and therapeutic effects of commonly used traditional Chinese botanical drugs on the condition. The focus is specifically on liver damage caused by alcohol-induced OS, excluding dietary botanical drugs.

The effective metabolites in traditional Chinese medicine are biologically active chemical metabolites that define the therapeutic potential of natural medicines, characterized by specific chemical structures and physicochemical properties. Botanical drugs are derived from medicinal plants through standardized processes that ensure quality and contain one or more active metabolites. Compared to traditional Chinese herbal formulas, botanical drugs from medicinal plants represent the integration of modern pharmaceutical technology with traditional medicine. In this processing method, herbs are processed into purified active metabolites through techniques such as filtration, evaporation, or other extraction methods. This approach not only serves as a means to discover new drugs but also enhances the understanding of traditional medicine. Therefore, using Chinese botanical drugs to treat alcoholic liver disease (ALD) is a viable method (Singh et al., 2015). Research has shown that various botanical drugs, including flavonoids, phenolics, alkaloids, terpenoids, saponins, and anthraquinones, exhibit preventive and therapeutic effects on alcohol-induced liver injury.

In the context of hepatitis, both during its acute and chronic phases, inflammatory cell-mediated secondary hepatocellular injury plays a pivotal role. Following ethanol-induced hepatocellular damage, sterile inflammatory mediators are released (Ashraf and Sheikh, 2015). Furthermore, an adaptive immune response mediated by B cells, T cells, and natural killer (NK) cells can precipitate a cytokine storm within the liver, leading to hepatocellular oxidative injury. The most significant period of inflammation in alcoholic liver disease (ALD) occurs during alcoholic hepatitis, which represents a relatively early stage of ALD pathogenesis (Reyes-Fermin et al., 2020). It is important to note that animal models for later stages such as hepatic fibrosis and cirrhosis are often induced pharmacologically rather than through alcohol exposure. Consequently, these models may not fully replicate the clinical progression observed in human patients. Moreover, clinical trials frequently have a paucity of participants representing advanced stages of the disease (Ajoolabady et al., 2023).

Therefore, the studies included in our literature review focus on animal or cellular models of acute and chronic alcoholic hepatitis, as well as patient cohorts diagnosed with alcoholic hepatitis, excluding those at more advanced stages of the disease. This approach ensures that the research findings are more directly applicable to the early inflammatory processes characteristic of ALD (Song et al., 2020).

**FIGURE 3:**
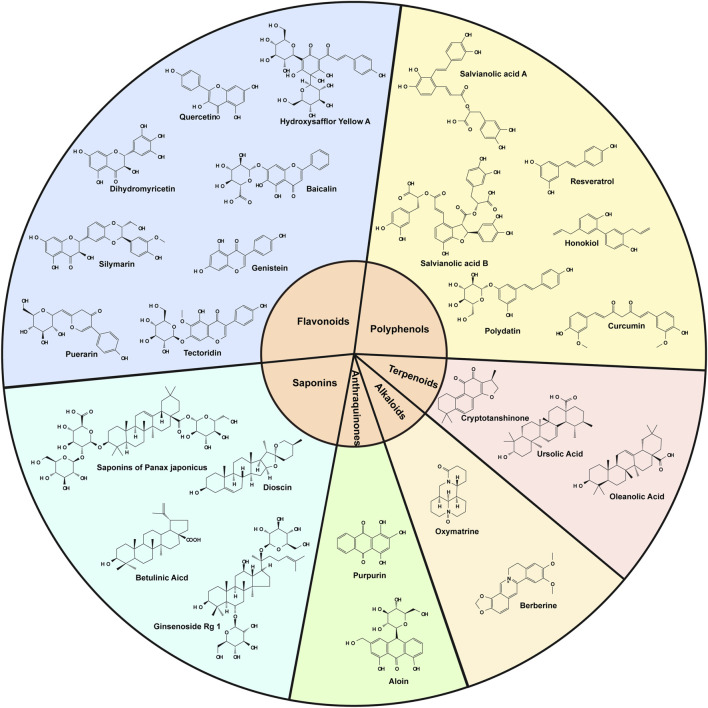
Diagrams of the chemical structures of all botanical drugs mentioned in the text.

This review summarizes the pharmacological mechanisms of selected botanical drugs in combating OS in ALD, offering a foundation for future studies on potential therapeutic agents for the treatment of alcoholic liver disease (Table 2). The chemical structures of the associated botanical drugs are shown separately in the figure (Figure 3).

### 7.1 Flavonoids: multitarget antioxidants and metabolic regulators

Flavonoids exert hepatoprotective effects through synergistic mechanisms that include direct ROS scavenging, activation of antioxidant pathways, and metabolic regulation. Their hydroxyl-rich structures enable electron donation to neutralize free radicals, while MAPK/Nrf2 signaling upregulates antioxidant enzymes such as SOD and GSH-Px (Ciumărnean et al., 2020; Singh et al., 2021). Additionally, flavonoids suppress SREBP-1c-driven lipogenesis and enhance PPAR-α-mediated β-oxidation, thereby alleviating hepatic lipid accumulation (Liu et al., 2021b).

Quercetin, a flavonol abundant in vegetables and medicinal herbs, activates the Nrf2/HO-1 pathway via MAPK/Nrf2 signaling to restore glutathione levels and reduce serum ALT/AST (Yao et al., 2007; Tang et al., 2012).

Puerarin, an isoflavone derived from the root of Pueraria montana var. lobata (Willd.) Maesen & S.M.Almeida ex Sanjappa & Predeep [Fabaceae], modulates AMPK/ACC pathways to alleviate hepatic TNF-α overexpression and steatosis (Zhou et al., 2014; Hu et al., 2023b).

Tectoridin, a key isoflavone metabolite found abundantly in the flowers of Pueraria montana var. lobata (Willd.) Maesen & S.M.Almeida ex Sanjappa & Predeep [Fabaceae], inhibits PPAR-α and mitochondrial dysfunction, preventing acute ethanol-induced hepatic steatosis (Xiong et al., 2010; Rong et al., 2023).

Genistein, an isoflavone from legumes such as soybeans and Trifolium pratense L. [Fabaceae], restores Nrf2 signaling to mitigate endoplasmic reticulum stress and hepatocyte apoptosis (Ding et al., 2023).

Hydroxysafflor Yellow A, a major metabolite of Carthamus tinctorius L. [Asteraceae], targets PI3K/Akt cascades to reduce mitochondrial ROS accumulation (Zhao et al., 2020; Wang et al., 2024).

Baicalin, a flavonoid metabolite extracted from the roots of Scutellaria baicalensis Georgi [Lamiaceae], suppresses the MicroRNA-205/NF-κB axis, attenuating inflammation and apoptosis (Yang et al., 2021b; Fang et al., 2022b).

Silymarin, a flavonoid from Silybum marianum (L.) Gaertn. [Asteraceae], inhibits NF-κB-driven cytokine storms and reduces fibrosis markers such as ALT and AST (Song et al., 2006; Kainan et al., 2018).

Dihydromyricetin (DHM), a bioflavonoid metabolite derived from plants such as Hovenia dulcis Thunb. [Rhamnaceae], enhances ethanol clearance via SIRT1/AMPK activation, restoring mitochondrial function (Silva et al., 2020; Janilkarn-Urena et al., 2023).

### 7.2 Polyphenols: orchestrators of redox balance and metabolic homeostasis

Polyphenols mitigate ALD by chelating iron to block Fenton reaction-driven hydroxyl radical generation, while epigenetic modulation through SIRT1-mediated deacetylation of NF-κB and histones further suppresses inflammation (Dini and Grumetto, 2022; Song et al., 2020). These metabolites also reprogram lipid metabolism by downregulating SREBP-1c and upregulating CPT-1-mediated fatty acid β-oxidation, thereby reducing hepatic lipid deposition (Nanji et al., 2003).

Honokiol, a polyphenol from Magnolia officinalis Rehder & E.H.Wilson [Magnoliaceae], inhibits SREBP-1c nuclear translocation, effectively reducing hepatic lipid accumulation (Yin et al., 2009).

Resveratrol, a non-flavonoid polyphenol from Veratrum album L. [Melanthiaceae], enhances nuclear SIRT1 translocation to ameliorate CYP2E1-induced mitochondrial dysfunction and oxidative damage (Peiyuan et al., 2017).

Polydatin, a resveratrol glycoside from plants like Reynoutria japonica Houtt. [Polygonaceae], lowers elevated ALT/AST levels and restores antioxidant balance following alcohol withdrawal (Lai et al., 2018; Lai, 2019).

Curcumin, the principal curcuminoid from Curcuma longa L. [Zingiberaceae], blocks Kupffer cell activation via NF-κB inhibition and replenishes hepatic GSH reserves (Nanji et al., 2003; Dong and Xu, 2017).

Salvianolic Acid A/B, water-soluble polyphenols from Salvia miltiorrhiza Bunge [Lamiaceae], augment ADH and ALDH2 activity, accelerating ethanol metabolism and reducing lipid droplet accumulation (Li et al., 2014; Zhang, 2024).

### 7.3 Terpenoids: Nrf2 activators and inflammation resolvers

Terpenoids combat ALD by activating the Nrf2 pathway to transcriptionally upregulate antioxidant genes such as HO-1 and SOD-1, thereby restoring redox balance (Liu et al., 2014). Concurrently, they suppress CYP2E1 activity, limiting acetaldehyde-induced ROS generation, and inhibit TLR4/MyD88 signaling to reduce TNF-α and IL-6 secretion (Nagappan et al., 2019; Zhao et al., 2019).

Oleanolic Acid, a pentacyclic triterpenoid metabolite isolated from plants such as Olea europaea L. [Oleaceae] and the fruits of Ligustrum lucidum W.T.Aiton [Oleaceae], induces Nrf2 nuclear translocation, enhancing antioxidant defenses in ethanol-challenged hepatocytes (Liu et al., 2014).

Ursolic Acid, a triterpenoid from the evergreen climbing shrub Arctostaphylos uva-ursi (L.) Spreng. [Ericaceae], attenuates DNA fragmentation and lipid peroxidation by inhibiting ROS accumulation (Zhao et al., 2019; Ma et al., 2021).

Tanshinones, the lipophilic active metabolites found in Salvia miltiorrhiza Bunge [Lamiaceae], a member of the Lamiaceae family. Classified as diterpenoids, these metabolites are sensitive to light (Estolano-Cobián et al., 2021). Cryptotanshinone, one of its metabolites, synergizes AMPK and SIRT1 activation to reverse SREBP-1c-driven lipogenesis and hepatic steatosis (Nagappan et al., 2019).

### 7.4 Alkaloids: suppressors of apoptosis and metabolic dysfunction

Alkaloids attenuate ALD by suppressing the MAPK/NF-κB axis, thereby reducing ERK/JNK phosphorylation and TNF-α production, which mitigates hepatocyte apoptosis (Huang et al., 2023). Independent of Nrf2, these metabolites elevate antioxidant enzymes such as CAT and GSH, further alleviating oxidative damage (Du et al., 2021).

Oxymatrine, a quinolizidine alkaloid from the roots of Sophora flavescens Aiton [Fabaceae], inhibits ERK1/2-mediated apoptosis and downregulates pro-inflammatory cytokines (Huan et al., 2023; Huang et al., 2023).

Berberine, an isoquinoline alkaloid from traditional Chinese medicinal herbs such as Coptis chinensis Franch. [Ranunculaceae] and Phellodendron chinense C.K.Schneid. [Rutaceae], restores the AMPK/SIRT1/PPAR-α axis, normalizing lipid turnover and reducing hepatic TG accumulation (Zhang et al., 2014).

### 7.5 Saponins: guardians of mitochondrial integrity and lipid peroxidation

Saponins protect against ALD by inhibiting CYP2E1 to reduce ROS generation during ethanol metabolism, while PPAR-α activation promotes fatty acid β-oxidation and HDL synthesis (Yang et al., 2021a; Moses et al., 2014). Additionally, they suppress the NLRP3 inflammasome, downregulating IL-1β and mitigating inflammatory responses (Qiu et al., 2023).

Dioscin, a natural steroidal saponin metabolite found in various herbs, including Dioscorea bulbifera L. [Dioscoreaceae] and Polygonum aviculare L. [Polygonaceae] (Bandopadhyay et al., 2022), enhances p62/Nrf2 interaction to boost transcription of antioxidant genes such as HO-1 (Moses et al., 2014).

Saponins of Panax japonicus, triterpene glycosides from Panax japonicus (T.Nees) C.A.Mey. [Araliaceae], activate the p62/Nrf2 pathway to suppress ROS and lipid peroxidation (Qiu et al., 2023).

Betulinic Acid (BA), also known as birch acid, is a pentacyclic triterpenoid saponin widely distributed in the plant kingdom, extracted from the leaves of Syzygium cumini (L.) Skeels [Myrtaceae], Betula pendula Roth [Betulaceae], and Ziziphus jujuba Mill. [Rhamnaceae] (Farzan et al., 2024). It can elevate SOD and CAT activity, effectively mitigating hepatic steatosis (Yi et al., 2014).

Ginsenoside Rg1, a protopanaxatriol-type saponin from Panax ginseng C.A.Mey. [Araliaceae] and Panax notoginseng (Burkill) F.H.Chen [Araliaceae], preserves mitochondrial membrane potential by inhibiting CYP2E1, thereby preventing cytochrome c release and apoptosis (Yang et al., 2021a).

### 7.6 Anthraquinones: dual modulators of lipogenesis and antioxidant defense

Anthraquinones alleviate ALD by suppressing the AMPK/SREBP-1c axis to inhibit *de novo* lipogenesis and activating the Nrf2/ARE pathway to upregulate antioxidant enzymes such as GST and GR (Cui et al., 2014; Hussain et al., 2022).

Purpurin, an anthraquinone from the roots of Rubia tinctorum L. [Rubiaceae], scavenges ROS and enhances antioxidant defenses via Nrf2 activation (Hussain et al., 2022).

Aloin, an anthraquinone glycoside from Aloe vera (L.) Burm. f. [Asphodelaceae], suppresses SREBP-1c maturation while activating AMPK to promote lipid catabolism (Cui et al., 2014).

## 8 Effect of exercise on oxidative stress

Exercise plays multiple roles in regulating systemic metabolic health, with mechanisms that include maintaining glucose homeostasis, optimizing lipid metabolism, and enhancing mitochondrial function (Park et al., 2023). Numerous clinical studies have shown that regular physical exercise can significantly improve insulin sensitivity by promoting the translocation of glucose transporter proteins to the cell membrane in skeletal muscle cells, thus enhancing glucose uptake and utilization in peripheral tissues (Liu et al., 2023). This effect is particularly important in preventing and alleviating insulin resistance commonly observed in ALD patients. In addition, exercise activates the AMPK signaling pathway, accelerating fatty acid β-oxidation, effectively reducing hepatic lipid accumulation, and improving blood lipid profiles, as evidenced by increased HDL levels and decreased LDL levels (Bekheit et al., 2025). Exercise also plays a positive role in alleviating ALD by breaking the vicious cycle between inflammation and oxidative stress. Mechanistic studies indicate that exercise-induced muscle contraction promotes the release of myokines (e.g., IL-6), which inhibit nuclear translocation of NF-κB in hepatic Kupffer cells (Figure 2) (Milkovic et al., 2023). More importantly, exercise significantly enhances hepatic mitochondrial energy metabolism, providing a new intervention target to correct metabolic disorders in ALD patients (Dempke and Reck, 2021).

ROS generated during exercise exhibit a characteristic dose-response relationship. Moderate exercise maintains ROS at normal physiological levels, where ROS act as key signaling molecules to activate the Nrf2 pathway and upregulate the expression of endogenous antioxidants such as HO-1, SOD, and GPx (Merry and Ristow, 2016). Preliminary animal studies show that exercise in high-fat diet mice resulted in reduced hepatic 4-HNE levels and upregulation of anti-apoptotic protein expression (Merry and Ristow, 2016). However, excessive exercise can lead to excessive accumulation of ROS, surpassing the body’s antioxidant defense capacity, thus inducing oxidative damage. Therefore, personalized exercise interventions are essential to prevent exercise-induced oxidative stress. Research shows that both moderate-intensity continuous training (MICT, 50%–70% VO_2_max) and high-intensity interval training (HIIT, 85%–95% VO_2_max) can effectively activate the Nrf2 pathway, with HIIT being 30%–40% more effective in upregulating antioxidant enzymes than MICT (Martinez-Canton et al., 2024).

Although both exercise and herbal medicine show significant efficacy in treating ALD individually, their potential synergistic effects when combined remain to be further explored. Current findings suggest that active components in herbal medicine (e.g., curcumin, resveratrol) activate the Nrf2 pathway by covalently modifying cysteine residues on Keap1 protein (Nosrati-Oskouie et al., 2022). Exercise activates this pathway through ROS-mediated mechanisms, and the two may have synergistic or additive effects (Tkaczenko and Kurhaluk, 2025).

## 9 Conclusion and future prospects

The pathogenesis of ALD is inextricably linked to dysregulation of OS-related signaling networks, involving dynamic interactions among key molecules such as SREBP, Nrf2, and AMPK. Bioactive metabolites in botanical drugs—flavonoids, alkaloids, saponins, and others—demonstrate therapeutic potential across disease stages through multi-tiered mechanisms, ranging from direct antioxidant defense to systemic regulation of metabolic-inflammatory balance.

The botanical drugs exhibit unique advantages due to their multi-target properties. Flavonoids (e.g., quercetin) and phenolic acids (e.g., salvianolic acid B) play pivotal roles in early-stage ALD by directly scavenging reactive oxygen species (ROS) and activating the Nrf2 pathway, thereby blocking lipid peroxidation and mitochondrial dysfunction (Li et al., 2015). As the disease progresses to the inflammatory phase, alkaloids (e.g., berberine) and terpenoids (e.g., tanshinone IIA) synergistically regulate lipid metabolism and inflammation through pathways such as AMPK/NF-κB and PPAR-α (Fang et al., 2022a; Yue et al., 2021). In advanced fibrotic stages, saponins (e.g., ginsenoside Rg1) and low-dose anthraquinones (e.g., emodin) delay collagen deposition by modulating the gut-liver axis and suppressing TGF-β1/Smad3 signaling (Yang et al., 2022).

Despite the promising therapeutic effects, we also recognize that the current studies have certain limitations that require improvement and refinement in future research.

Firstly, the studies included in this review are primarily based on animal models and few human patients of acute or chronic alcoholic hepatitis. While these models provide preliminary insights into the mechanisms of action of herbal extracts, they fall short in distinguishing the specific stages of ALD. Future research should focus more on characterizing different stages of ALD to evaluate the effects of herbal extracts at specific disease stages, thereby providing a stronger theoretical foundation for clinical applications.

Secondly, we have not yet involved direct comparisons between traditional Chinese herbal medicines and other clinical drugs. Such comparative trials are crucial for comprehensively assessing the efficacy of herbal extracts. To better understand the advantages and limitations of herbal treatments relative to existing therapies, future research should actively incorporate such comparative experiments, offering more reliable data support for clinical decision-making.

Furthermore, while we have gained some understanding of the functions and mechanisms of biomolecules, many areas remain unexplored. For instance, the detailed functions and precise mechanisms of biomolecules involved in oxidative stress need further elucidation. Additionally, potential signaling pathways such as SIRT1, PI3K/Akt, and gene expression changes warrant deeper investigation.

To ensure the consistency and reproducibility of experimental results, standardization of herbal extracts is critical. Given that the chemical composition of herbal extracts can vary due to different processing methods, and that different extracts may share similar biological functions and molecular structures, it is essential to adopt standardized research methods and experimental models in study design. Increasing sample sizes and considering potential confounding factors can enhance the reliability of experimental results and lay a solid foundation for future clinical applications. Modern analytical methods can be employed to study the chemical characteristics of herbal extracts, further improving the robustness of the findings.

Regarding drug dosing and safety, high doses may lead to toxicity, while low doses may not achieve sufficient therapeutic effects. Data on optimal dosing and safety profiles of herbal extracts are relatively limited in past studies. Future research should pay greater attention to dose selection, determining appropriate dose ranges based on animal experiments and pharmacokinetic studies, and documenting any adverse effects. Considering individual differences and potential safety risks, adjusting doses appropriately is crucial for ensuring both the safety and efficacy of treatment. Moreover, long-term safety should be explored to assess the feasibility of using herbal extracts as a long-term therapeutic option.

Furthermore, this study not only systematically elucidates the mechanisms by which active components of Chinese botanical drugs alleviate alcohol-related liver disease (ALD) via modulation of oxidative stress, but also reveals the convergence of molecular targets between exercise interventions and botanical drug therapies. Both approaches commonly act on key signaling pathways, including Nrf2, NF-κB, and AMPK, indicating a potential for synergistic interaction. Future studies should focus on developing combinatorial strategies, such as identifying optimal pairings of exercise modalities (e.g., high-intensity interval training versus moderate-intensity endurance training) with specific classes of Chinese herbal medicine, elucidating the cross-regulation mechanisms within the Nrf2/NF-κB/AMPK signaling network, and establishing individualized intervention protocols that integrate exercise with botanical treatment for ALD patients.

This direction is particularly relevant to the field of sports medicine, where exercise-induced metabolic adaptations could potentiate the hepatoprotective effects of plant-derived compounds. For instance, enhanced hepatic perfusion resulting from aerobic activity may facilitate the targeted delivery and absorption of active phytochemicals, while exercise-induced ROS generation may synergize with polyphenol-mediated Nrf2 activation to upregulate endogenous antioxidant systems. Moreover, sustained metabolic improvements triggered by regular physical activity could help maintain the therapeutic benefits of botanical drugs even after cessation of treatment.

These synergistic effects provide a promising theoretical foundation for the development of integrated therapeutic approaches combining Chinese herbal medicine with personalized exercise interventions, offering a novel and practical strategy for the management of ALD within both clinical and rehabilitative contexts.
